# Perinatal and prenatal alcohol exposure impairs striatal cholinergic function and cognitive flexibility in adult offspring

**DOI:** 10.1016/j.neuropharm.2025.110627

**Published:** 2025-08-06

**Authors:** William Purvines, Himanshu Gangal, Xueyi Xie, Joseph Ramos, Xuehua Wang, Rajesh Miranda, Jun Wang

**Affiliations:** a Department of Neuroscience and Experimental Therapeutics, College of Medicine, Texas A&M University Health Science Center, Bryan, TX, 77807, United States; b Texas A&M Institute for Neuroscience, Texas A&M University, College Station, TX, 77843, United States

**Keywords:** Fetal Alcohol Spectrum Disorder (FASD), Cholinergic interneurons, Behavioral flexibility, Compulsive drinking

## Abstract

Fetal Alcohol Spectrum Disorder (FASD), caused by perinatal alcohol exposure (PeAE) and prenatal alcohol exposure (PAE), is characterized by significant cognitive impairments, including reduced cognitive flexibility. Despite the critical role of cholinergic interneurons (CINs) in the dorsomedial striatum (DMS) for cognitive and behavioral flexibility, their contribution to neurobehavioral deficits in FASD remains unclear. To address this gap, this research explored the impact of PeAE and PAE on CIN populations and activity, cognitive flexibility, and compulsive drinking behaviors in adult offspring. We first assessed CIN number by staining striatal sections for choline acetyltransferase (ChAT) and observed a significant reduction in CINs within the posterior striatum of adult PeAE offspring. Functional assessments revealed that PeAE and PAE markedly decreased CIN firing activity and reduced acetylcholine (ACh) release in the DMS, as measured by electrophysiology recordings and live-tissue confocal imaging using a genetically encoded ACh sensor. Behaviorally, PeAE offspring exhibited a significant deficit in adapting to reversed action-outcome contingencies despite intact initial learning capabilities. Moreover, PeAE mice exhibited compulsive alcohol drinking behavior, characterized by elevated consumption and preference for quinine-adulterated alcohol. These findings collectively highlight the critical role of impaired cholinergic signaling in the cognitive and behavioral deficits observed following PeAE and PAE. Understanding this cholinergic dysfunction provides valuable insights necessary for developing targeted interventions aimed at mitigating cognitive and behavioral consequences associated with FASD.

## 1. Introduction

Fetal Alcohol Spectrum Disorder (FASD) encompasses a continuum of neurodevelopmental and neuropsychiatric conditions caused by perinatal alcohol exposure (PeAE) and prenatal alcohol exposure (PAE), affecting an estimated 1.1–9.8% of the U.S. population in school systems (May et al., 2014; May et al., 2018). Individuals with FASD exhibit persistent impairments in cognition, motor function, and brain structure that can extend into adulthood. Among the various cognitive deficits, impaired cognitive flexibility, the capacity to adapt behavior in response to changing environmental contingencies, is a prominent symptom that contributes to impaired executive function (Green et al., 2009; Mattson et al., 1999; McGee et al., 2008). However, despite its clinical salience, the underlying neural mechanisms responsible for this impairment remain poorly understood.

The dorsomedial striatum (DMS) has emerged as a key neural substrate supporting cognitive flexibility (Bradfield et al., 2013; Huang et al., 2024; Ma et al., 2022; Ragozzino et al., 2002). Furthermore, the DMS is vulnerable to the effects of developmental alcohol exposure, with previous studies reporting PAE-induced alterations in striatal plasticity and medium spiny neuron (MSN) function (Cheng et al., 2018; Roselli et al., 2020). Within the DMS, MSNs form the principal output of the striatum, while cholinergic interneurons (CINs), though sparse in number, play a critical regulatory role in shaping striatal circuit dynamics (Gerfen and Surmeier, 2011; Kreitzer, 2009; Kreitzer and Mal- enka, 2008). CINs are the primary source of acetylcholine (ACh) in the striatum and exert widespread modulatory control over striatal function (Huang et al., 2024; Gangal et al., 2023; Ma et al., 2022). DMS CINs, whose dynamic firing is shaped by upstream excitatory inputs, are essential for behavioral flexibility, as CIN circuit manipulation alters reversal learning (Bradfield et al., 2013; Li et al., 2025; Ma et al., 2022; Ragozzino et al., 2009). Notably, gestational alcohol exposure has been shown to impair CIN excitability in the dorsolateral striatum (Bariselli et al., 2023). However, the impact of PeAE and PAE on CINs within the DMS and the behavioral consequences of such alterations remain largely unexplored. Given the established role of DMS CINs in mediating cognitive flexibility, this is a critical knowledge gap.

Our prior work has demonstrated that alcohol-induced CIN dysfunction in the DMS impairs behavioral flexibility in adult rodents (Gangal et al., 2023; Huang et al., 2024; Ma et al., 2022), raising the possibility that similar mechanisms may underlie cognitive deficits seen following PeAE and PAE. In this study, we used preclinical mouse models of FASD by exposing pregnant dams to PeAE via two-bottle choice drinking (2BC) or PAE via alcohol vapor inhalation. We found that PeAE reduced the population of DMS CINs and suppressed their activity. PAE also decreased CIN firing and impaired ACh release in the DMS of adult offspring. These cellular deficits were accompanied by marked impairments in cognitive flexibility seen in PeAE mice, as evidenced by failure to adapt to reversed action-outcome contingencies, and by increased compulsive drinking of quinine-adulterated alcohol. Our findings suggest that CIN dysfunction in the DMS could be a key neural mechanism underlying behavioral inflexibility and compulsive alcohol-seeking behavior following PeAE and PAE. These results not only expand our understanding of FASD pathophysiology but also highlight DMS cholinergic circuits as a promising therapeutic target for mitigating cognitive deficits associated with developmental alcohol exposure.

## 2. Materials and methods

### 2.1. Animals

ChAT-Cre, Ai14, and Ai32 mice were obtained from the Jackson laboratory. Tail DNA samples were collected from mice and PCR was conducted to determine genotype. Mice were housed in same-sex colonies under a 12h light/dark schedule, with food and water available ad libitum. All animal procedures in this study were approved by the Texas A&M University Institutional Animal Care and Use Committee. All procedures were conducted in agreement with the Guide for the Care and Use of Laboratory Animals, National Research Council, 1996.

### 2.2. Breeding and peri-/prenatal alcohol exposure

To comprehensively assess the impact of developmental alcohol exposure across distinct time windows and delivery methods, we employed both a voluntary drinking paradigm (PeAE) and a vapor inhalation paradigm (PAE), each selected for their specific translational and experimental strengths.

For experiments investigating the effect of PeAE on offspring, female mice were trained to consume high levels of alcohol for 6 weeks using the intermittent access to 20% alcohol two-bottle choice (2BC) procedure and were then mated with alcohol-naïve male mice as previously described (Cheng et al., 2018; Gangal et al., 2023; Huang et al., 2024; Iannucci et al., 2023; Ma et al., 2022; Wang et al., 2023; Xie et al., 2023). This paradigm allowed us to study how voluntary drinking, a model of human drinking, affects DMS CINs and behavioral outcomes in PeAE offspring. In this procedure (Supplementary Fig. 1A), mice were given 24-hr access to 1 bottle of 20% alcohol in water (vol/vol) and 1 bottle of water every other weekday (Monday, Wednesday, Friday). Alcohol solutions were prepared by diluting 200-proof pure ethanol in drinking water. Bottle placement was altered each session to control for side preference. Using this protocol, we have previously observed that pregnant dams reach alcohol intake levels of 20–30 g/kg over 24 h (Cheng et al., 2018). Such intake has been previously shown to induce blood alcohol concentrations (BACs) approaching 100 mg/dL (Hwa et al., 2011). For the control (Ctrl) group, female mice were given free access to water only. During mating, only water was administered to prevent males from drinking. After mating, impregnated females were re-exposed to alcohol using the 2BC procedure until postnatal day 10. PeAE and Ctrl offspring were separated by sex at 21 days old and housed in groups of 3–5. Gestational day 0 (GD0) was approximated as the halfway point of the 24-h mating session (12 h after males were introduced).

We complemented our PeAE paradigm with a prenatal alcohol vapor inhalation model in Figs. 2 and 3 to asses how alcohol exposure confined to prenatal development affects DMS cholinergic function. Using another mouse model of FASD also allowed us to assess the generalizability of PeAE-driven alterations in CIN function. This PAE model (Supplementary Fig. 1B) was utilized as alcohol vapor inhalation is known to achieve higher, more reliable blood alcohol concentrations (BACs) compared to voluntary drinking models which often produce lower, more variable BACs (Gilpin et al., 2008). Moreover, we selected the vapor inhalation model to precisely target a critical neuro- developmental window (fetal days 11–15), enabling us to determine more direct effects of alcohol on CINs. Pregnant dams were either exposed to air or alcohol vapor pumped into a plexiglass chamber (92 x 62 × 36 cm) for 1.5–2 h on fetal days 11–15 to produce Ctrl and PAE offspring, respectively. Our vapor inhalation set up was adapted from (Morton et al., 2014) and has been previously described (Vierkant et al., 2023). To produce alcohol vapor, air was pumped into a sealed flask containing 200 proof alcohol via an aeration stone, which was then mixed with air before entering the chamber. In trial experiments, this vapor exposure induced BACs of ~120 mg/dL. After pups were weaned, they were separated and housed as described in the 2BC procedure. GD0 was estimated as the middle of the 48-h mating period (24 h after males were introduced).

### 2.3. Histology

Mice were deeply anesthetized with isoflurane and then perfused with 4% paraformaldehyde (PFA) in phosphate-buffered saline (PBS). Brains were collected and submerged in 4% PFA/PBS solution overnight at 4 °C before being transferred to 30 % sucrose in PBS. After brains sunk, they were sliced on a cryostat into 50 μm sections. Sections were stained with anti-ChAT antibody (AB144P) followed by a secondary antibody conjugated to a 647 nm-emmiting fluorophore (A21447, Alexa-Fluor 647) (Essoh et al., 2025; Gangal et al., 2023). Confocal images were obtained with an Olympus Fluoview 1200 microscope using a 10× objective lens. Images were stitched with the same program and fluorescent neurons were counted using Imaris.

### 2.4. Electrophysiology

Electrophysiological recordings were conducted as previously described (Essoh et al., 2025; Gangal et al., 2023; Huang et al., 2024; Ma et al., 2022; Wang et al., 2011). Mice were deeply anesthetized with isoflurane before perfusion and brain extraction. DMS-containing slices (250 μM) were then collected in ice-cold cutting solution. Cutting solution contained (in mM): 40 NaCl, 148.5 sucrose, 4 KCl, 1.25 NaH_2_PO_4_, 25 NaHCO_3_, 0.5 CaCl_2_, 7 MgCl_2_, 10 glucose, 1 sodium ascorbate, 3 sodium pyruvate, and 3 myo-inositol, saturated with 95 % O_2_ and 5 % CO_2_. Slices were incubated in a 1:1 mixture of cutting and external solution held at 32 °C for 45 min before being transferred to pure external solution at room temperature for 15 min before use and the duration of the experiment. External solution consisted of (in mM): 125 NaCl, 4.5 KCl, 2.5 CaCl_2_, 1.3 MgCl_2_, 1.25 NaH_2_PO4, 25 NaHCO_3_, 15 sucrose, and 15 glucose, saturated with 95 % O_2_ and 5 % CO_2_.

In the recording chamber, slices were perfused with 32 °C external solution at a flow rate of 1.5–2 mL/min. CINs were identified by their Cre-driven expression of fluorescent reporter and their large soma size. All recordings were conducted using a Multiclamp 700B amplifier controlled by pClamp 11.4 software (Molecular Devices). In both whole-cell patch clamp and cell-attached recordings, a K^+^ intracellular solution was used, consisting of (in mM): 123 potassium gluconate, 10 HEPES, 0.2 EGTA, 8 NaCl, 2 MgATP, 0.3 NaGTP (pH 7.3), with an osmolarity of 270–280 mOsm.

To measure excitability in whole-cell patch clamp recordings, CINs were recorded in current-clamp mode and action potentials were evoked with stepped current injections at 25-pA increments lasting 500ms. CIN spontaneous and burst-pause firing was measured using cell-attached recording in voltage clamp mode. For optogenetic excitation in burstpause recordings, 10, 15, and 20 Hz blue light stimulation (473 nm, 2 ms pulse width) was delivered through the objective lens.

### 2.5. Live tissue confocal imaging of ACh release

A green ACh sensor, AAV-gACh4m (Huang et al., 2024; Jing et al., 2020), was bilaterally infused into the DMS of ~4 month old WT PAE or Ctrl mice (AP: 0.1, ML: ±1.87, DV: 2.9 mm). Viral infusion took place on a stereotaxic frame and mice were anesthetized with 3–4 % isoflurane at a flow rate of 1.0 L/min during the entire procedure. 2 weeks later, acute brain slices were collected in the same manner as in electrophysiology experiments. Slices were held in a recording bath continuously perfused with external solution saturated with 95 % O_2_ and 5 % CO_2_. ACh release was imaged with an Olympus Fluoview FV3000 microscope using a 40x NA 0.8 water immersion objective along with a 488 nm and 561 nm laser. A sample rate of ~2 frames per second was used for imaging, and all imaging parameters were kept consistent throughout all imaging sessions. MATLAB scripts were used to extract time-course ΔF/F values to quantify spontaneous event frequency and release amplitude.

### 2.6. Instrumental reversal learning

The instrumental reversal learning procedure was adapted from (Bradfield et al., 2013) and conducted as previously described (Gangal et al., 2023; Huang et al., 2024; Ma et al., 2022). Training began with either a purified pellet or grain pellet sub-session. The purified pellets (Bio-Serv, F06233) are nutritionally defined and primarily composed of sucrose, dextrose, and casein, while the grain pellets (Bio-Serv, F0163) contain a standard mix of grain-derived ingredients. These pellets were selected for their distinct taste, allowing for clear discrimination and effective outcome devaluation using rewards of equal mass (Gangal et al., 2023; Matamales et al., 2020). During these sessions, the house light was on, and one of the levers was presented. Initially, pressing the left lever (A1) resulted in delivery of a grain pellet reward (O1), while pressing the right lever (A2) delivered a purified pellet reward (O2). PeAE and Ctrl mice were first trained under a fixed ratio 1 (FR1) schedule for three days, where each lever press yielded one reward. The mice were then transitioned to a random ratio (RR) schedule, beginning with RR5 (probability of reward = 0.2 per press) for three days, then progressing to RR10 for another three days, and finally progressing to RR20 for two days. The initial devaluation test occurred one day after the final RR20 training session. Prior to the test, animals had 1-h free access to 40 pellets (randomly assigned as either purified or grain) to induce satiety. The test began either 10 min after consuming all the pellets or after a maximum of 60 min. During the 10-min session, both levers were presented, but lever presses did not result in reward delivery. The lever corresponding to the pre-fed pellet type was classified as devalued (Dev), while the lever associated with the pellet type not pre-fed was classified as valued (Val). The test procedure was repeated with the alternative pellet type on the following day. Val and Dev data points were calculated as the sum of responding for each valued reward and devalued reward across the two sessions, respectively. The devalulation index was calculated as (Val−Dev)/(Val + Dev).

Mice were then exposed to four sessions of reversal learning. During reversal, action-outcome contingencies were switched: the left lever (initially A1→O1 paired with grain pellet O1) now delivered the purified pellet reward (A1→O2), and the right lever (initially A2→O2 paired with purified pellet O2) delivered grain pellet reward (O1). Another devaluation test was then conducted to asses how well mice learned the reversed contingencies. This second devaluation test followed the same procedure as the initial devaluation test.

### 2.7. Intermittent access two-bottle choice drinking procedure

2BC drinking was conducted as previously described (Cheng et al., 2017, 2018; Gangal et al., 2023; Ma et al., 2022; Vierkant et al., 2023; Xie et al., 2023). Adult PeAE and Ctrl mice were individually housed to assess alcohol drinking. Mice were presented with two bottles: one containing a 20% alcohol solution in drinking water and the other containing drinking water only, provided three times a week (Monday, Wednesday, Friday) for 24 h. Both sets of bottles were replaced with fresh solutions weekly. To measure alcohol consumption, bottles were weighed before and after each session, and alcohol intake was calculated as the grams of alcohol consumed divided by the mouse’s body weight in kilograms (g/kg/24h). Alcohol preference was determined as the total alcohol solution intake divided by the total fluid intake, multiplied by 100%. To account for drippage and evaporation, additional water and alcohol bottles were placed over empty cages during sessions. In experiments examining compulsive drinking behavior, quinine hemisulfate salt was added to the 20% alcohol solution at a concentration of 50 mg/L.

### 2.8. Statistical analysis

**Fig. 1.**
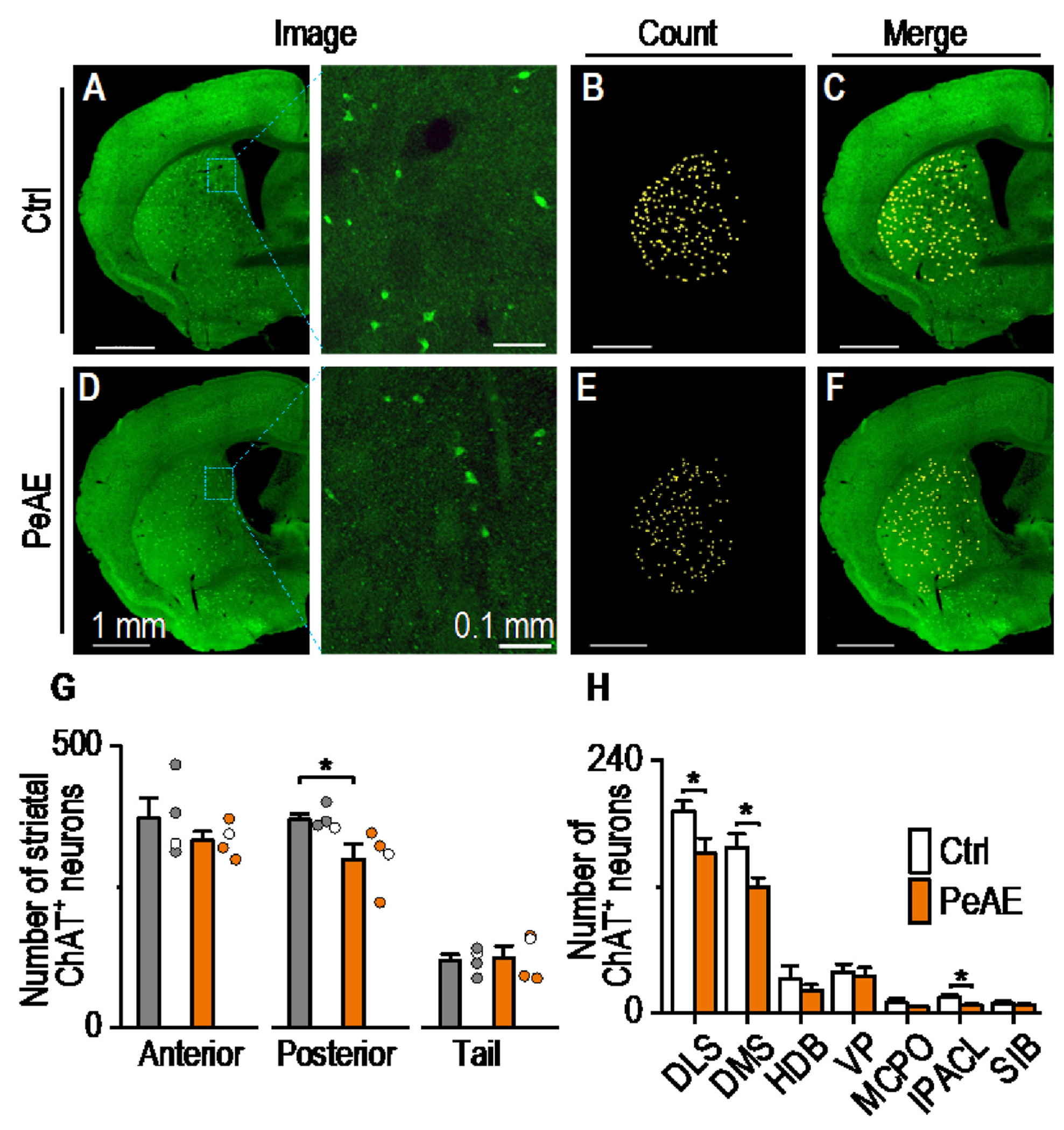
PeAE reduces cholinergic interneuron numbers in the posterior striatum. ChAT-Cre; Ai14 mice were exposed to perinatal alcohol (PeAE) or water (Ctrl), and coronal brain sections were obtained and stained with an anti-ChAT antibody at ~8 months of age. ***A-F***, Sample images from posterior sections showing ChAT-immunoreactive (ChAT^+^) neurons (*A*, *D*), spots of counted ChAT^+^ neurons (*B*, *E*), and merged images (*C*, *F*) in sections from control (Ctrl, *A*-*C*) and perinatal alcohol exposed (PeAE, *D*-*F*) mice. Enlarged images in *A* and *D* show fewer ChAT ^+^ neurons in the DMS of a PeAE mouse (*D*) than in that of a Ctrl mouse (*A*). ***G***, Bar graph comparing ChAT^+^ neuron counts in anterior (left, 1.70 to 0.62 mm), posterior (middle, 0.50 to −0.46 mm), and tail (right, −0.58 to −2.06 mm) brain sections. Group × session interaction, *p* = 0.057 by 2-way RM ANOVA with Tukey’s post-hoc tests. Post-hoc test results: Group within the anterior plane, *p* = 0.235; group within the posterior plane, *p* = 0.043; group within the tail of striatum *p* = 0.841, *p < 0.05. The 4 Ctrl mice (3 females, 1 male) were derived from 3 litters and the 4 PeAE mice (3 females, 1 male) were derived from 4 litters. Filled symbols are female data points and open symbols are male data points. ***H***, Bar graph comparison of tdT^+^ neurons between Ctrl and PeAE mice across multiple brain regions. Unpaired *t*-test results: DLS, *p* = 0.044; DMS, *p* = 0.039, IPACL, *p* = 0.040, *p < 0.05. n = 5 mice per group. The 5 Ctrl mice (3 females, 2 males) were derived from 4 litters and the 5 PeAE mice (3 females, 2 males) were derived from 5 litters. DLS, dorsolateral striatum; VP, ventral pallidum; HDB, horizontal diagonal band; IPACL, interstitial nucleus of the posterior limb of the anterior commissure lateral part; MPCO, magnocellular preoptic nucleus; SIB, Substantia innominata-basal part.

Data from male and female subjects were combined for analysis and sex differences were not evaluated. All data were analyzed using either unpaired *t* tests or two-way ANOVA with repeated measures (2-way RM ANOVA), followed by Tukey’s *post-hoc test*. Statistical analysis was conducted using SigmaPlot or SPSS. Data in all figures was analyzed using the individual animal as the experimental unit. Statistical analysis using litter as the experimental unit for Figs. 1 and 4 and 5 has also been included in Supplementary Table 1. When data did not follow normal distribution, Generalized Linear Mixed Model (GLMM) tests were conducted to assess main effects and interactions, and Mann-Whitney Sum tests were used when data for unpaired t-tests was not normally distributed. All data are expressed as the Mean ± SEM.

### 2.9. Blinding and randomization

**Fig. 2.**
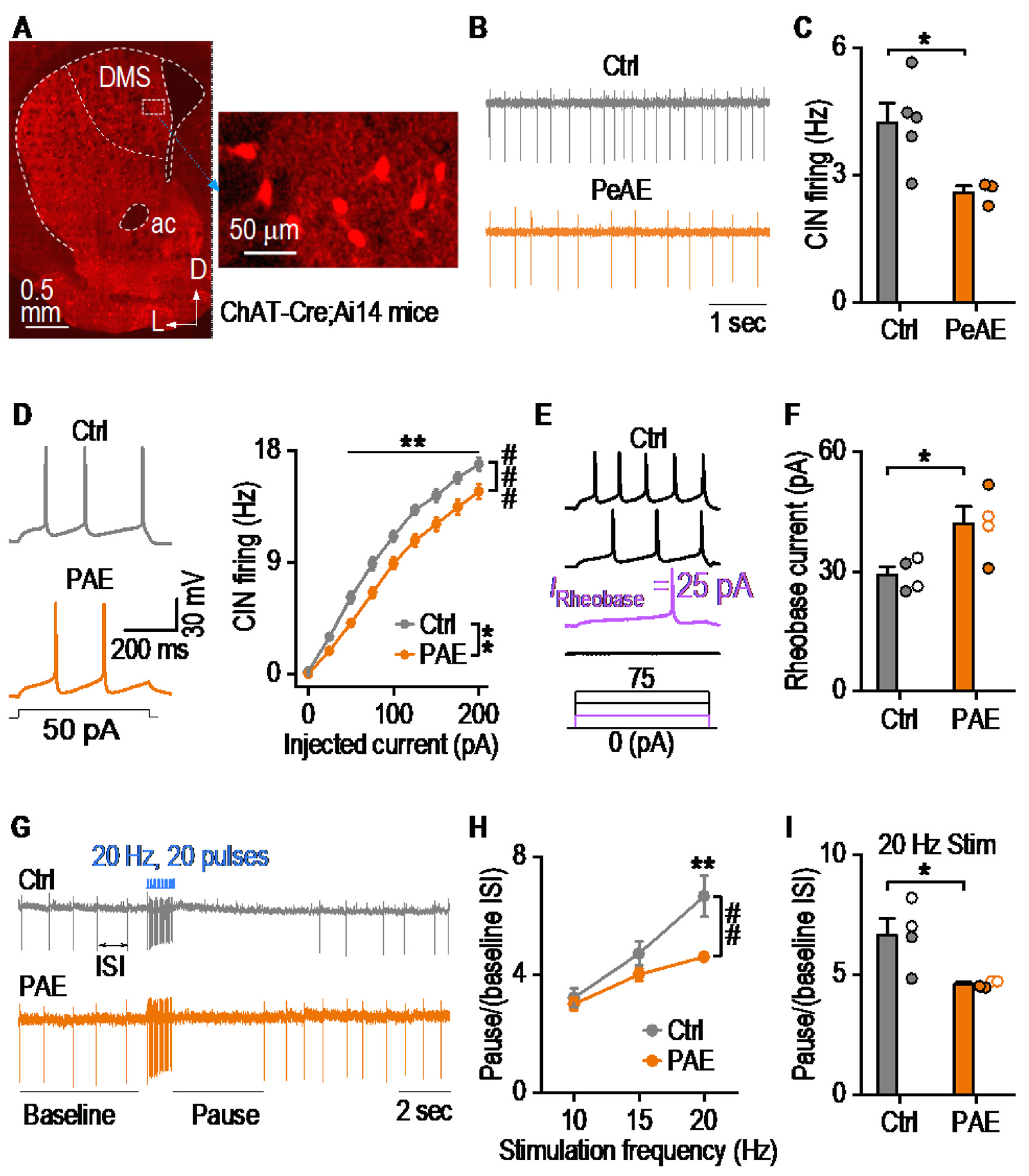
PeAE and PAE reduce CIN firing activity in the DMS of adult offspring. ***A***, Representative image of a ChAT-Cre; Ai14 mouse showing tdT expression in CINs of the DMS. ac, anterior commissure. Scale bar: 50 μm (inset). ChAT-Cre; Ai14 mice were exposed to perinatal alcohol or water. DMS slices were collected at 8 months of age. ***B***, Representative traces of spontaneous CIN firing in Ctrl and PeAE groups recorded using cell-attached configuration. ***C***, PeAE significantly decreases the spontaneous firing rate of DMS CINs in adult offspring. *p* = 0.039 by unpaired *t*-test, *p < 0.05. n = 5 mice from 5 litters for Ctrl and 3 mice from 3 litters for PeAE. All mice are female, and we recorded 3–5 neurons per mouse. ***D***, ChATCre; Ai32 mice were exposed to air (Ctrl) or alcohol vapor (PAE) prenatally and recorded at ~5 months of age. PAE significantly reduced evoked firing of DMS CINs in response to step-current injection. Left, sample traces of evoked CIN firing in Ctrl and PAE groups. Right, CINs from PAE offspring fired significantly less in response to current injection compared to Ctrl. Group effect, *p* = 0.008; Group x stimulation effect *p* < 0.001, **p < 0.01, ###*p* < 0.001 by 2-way RM ANOVA with Tukey’s post-hoc tests. n = 4 mice per group. Each group contains 2 males and 2 females. We recorded 6–14 neurons per mouse. ***E***, Representative trace illustrating the rheobase current in a CIN from a Ctrl animal. ***F***, PAE mice exhibited higher rheobase currents than Ctrl animals. Rheobase current was defined as the first current step, within a series of +25 pA steps beginning at 0 pA, capable of eliciting one action potential. *p* = 0.037 by unpaired *t*-test, *p < 0.05. n = 4 mice per group. Each group contains 2 males and 2 females. Filled symbols are female data points and open symbols are male data points. We recorded 6–14 neurons per mouse. ***G***, Representative traces from a Ctrl and PAE mouse showing burst-pause responses in CINs evoked by optogenetic stimulation. ***H***, PAE significantly reduced normalized pause durations compared to Ctrl mice. The pauses were induced by escalating frequencies of optogenetic stimulation (each lasting 1 s). The normalized pause duration was calculated by dividing the raw pause duration by the average baseline inter-spike interval (ISI) prior to stimulation. Group × stimulation interaction, *p* = 0.003 by 2-way RM ANOVA with Tukey’s post-hoc tests, ##*p* < 0.01, **p < 0.01. n = 4 mice per group. Each group contains 2 males and 2 females. We recorded 2–5 neurons per mouse. ***I***, Comparison of normalized pause duration following 20 Hz stimulation between Ctrl and PAE mice. CINs from PAE mice exhibited significantly shorter normalized pause durations than those from Ctrl mice. *p* = 0.026 by unpaired *t*-test, *p < 0.05. n = 4 mice per group. Each group contains 2 males and 2 females. Filled symbols are female data points and open symbols are male data points. We recorded 2–5 neurons per mouse. All statistics were conducted using animal means.

**Fig. 3.**
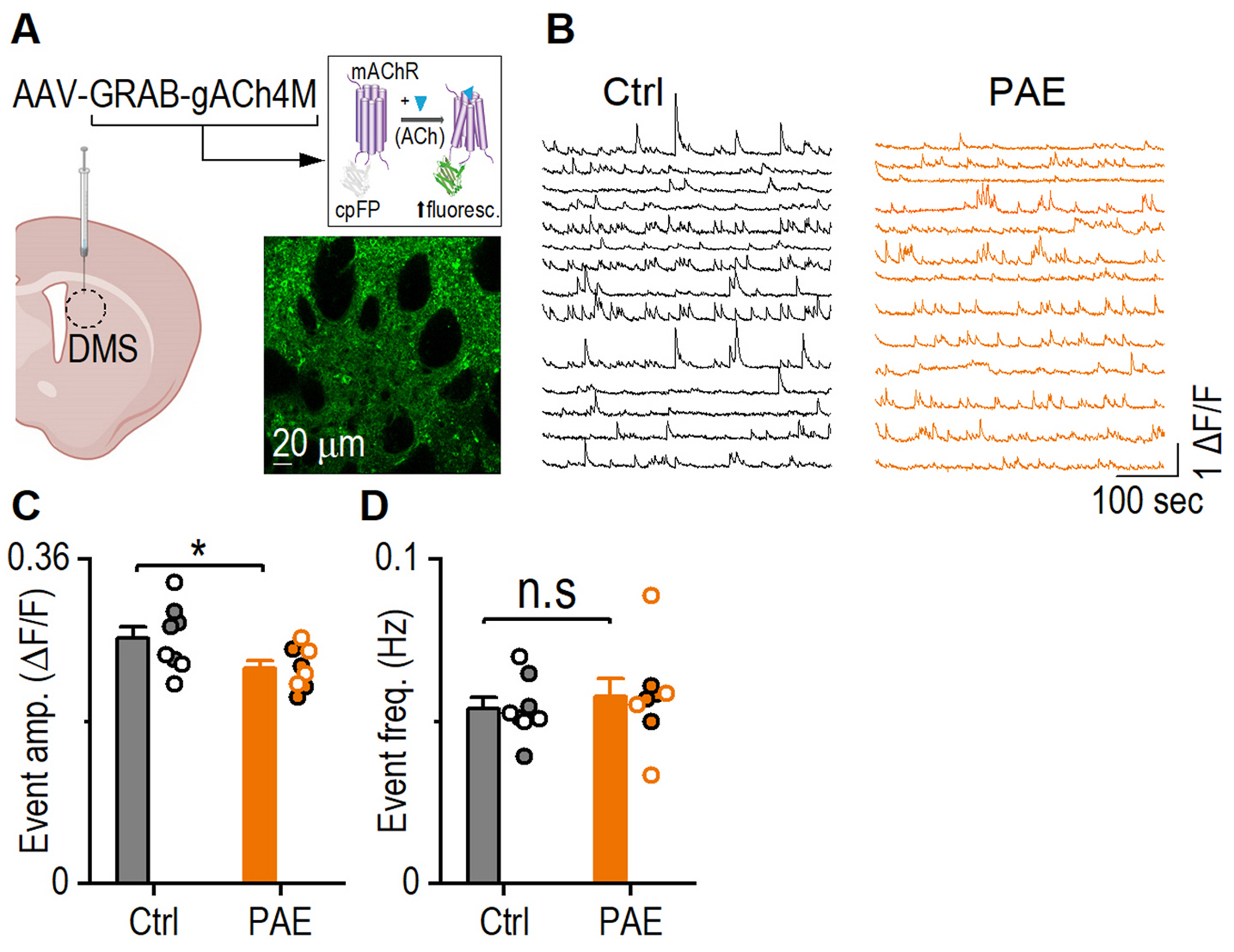
PAE reduces ACh release in the DMS of adult offspring. ***A***, Schematic illustrating viral infusion of AAV-gACh4m into the DMS (left) and a representative image showing the expression of the ACh sensor GRAB-ACh4m (right). Pregnant C57BL/6 dams were exposed to air (Ctrl) or alcohol vapor (PeAE) during embryonic days 11–15. At ~4 months of age, offspring from each group received DMS infusions of AAV-gACh4m. DMS slices were prepared 2 weeks later for live confocal imaging. ***B***, Representative traces of ACh release events recorded via live-tissue confocal imaging in Ctrl and PAE groups. ***C,*** PAE significantly reduced the amplitude of ACh release events in DMS slices from adult offspring. *p* = 0.047 by unpaired *t*-test, *p < 0.05. ***D,*** PAE did not alter the frequency of ACh release events. *p* = 0.57 by unpaired *t*-test. n = 8 mice (4 females, 4 males) from 3 litters (Ctrl) and 8 mice (4 females, 4 males) from 2 litters (PAE). Filled symbols are female data points and open symbols are male data points.

**Fig. 4.**
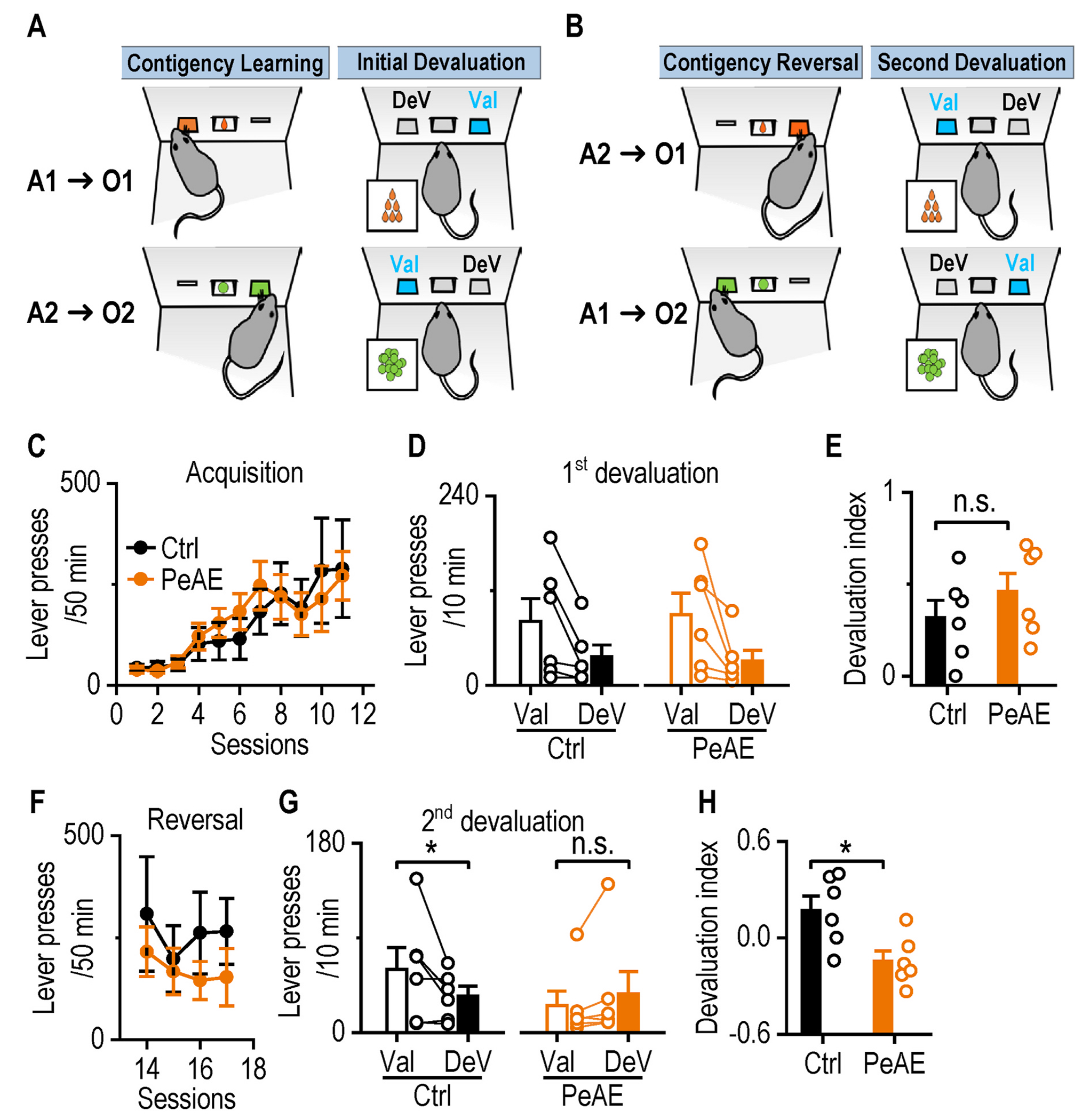
PeAE impairs reversal learning of instrumental conditioning in adult offspring. ***A***, Schematic of initial action-outcome (A–O) contingency learning in an operant task. Mice aged ~8 months old exposed to perinatal water (Ctrl) or alcohol (PeAE) underwent 11 training sessions to press the left lever (A1) for a grain pellet reward (O1) and the right lever (A2) for a purified pellet reward (O2). A devaluation test followed, where each reward was pre-fed before an operant test (devalued) to assess A-O contingency learning. Val, valued; Dev, devalued. Val and Dev data points are the sum of lever presses for the valued and non-valued levers, respectively. ***B,*** Schematic of reversal learning, where A1 was now paired with O2 and A2 with O1. A second devaluation test assessed the learning of reversed A-O contingencies. ***C***, Initial training lever press rates were similar between PAE and Ctrl mice. Group × session interaction, *p* = 0.80 by GLMM. ***D***, Both groups were sensitive to outcome devaluation following initial training. Group x session, *p* = 0.635 by GLMM; Effect of session on all subjects, *p* = 0.005 by GLMM. ***E***, During the first devaluation test, PeAE did not affect the devaluation index, calculated as (Val−Dev)/(Val + Dev). *p* = 0.32 by unpaired *t*-test, n.s., not significant. ***F***, Lever pressing rates during reversal learning training were comparable between groups. ***G***, PeAE mice exhibited reduced sensitivity to outcome devaluation after reversal learning compared to Ctrl mice. Group × session interaction, *p* = 0.037 by 2-way RM ANOVA with Tukey’s post-hoc tests, *p < 0.05. n.s., not significant. ***H***, The devaluation index was significantly lower in PeAE mice compared to Ctrl mice during the second devaluation test. *p* = 0.016 by unpaired *t*-test, *p < 0.05. n = 6 male mice from 4 litters (Ctrl) and 6 male mice from 4 litters (PeAE).

**Fig. 5.**
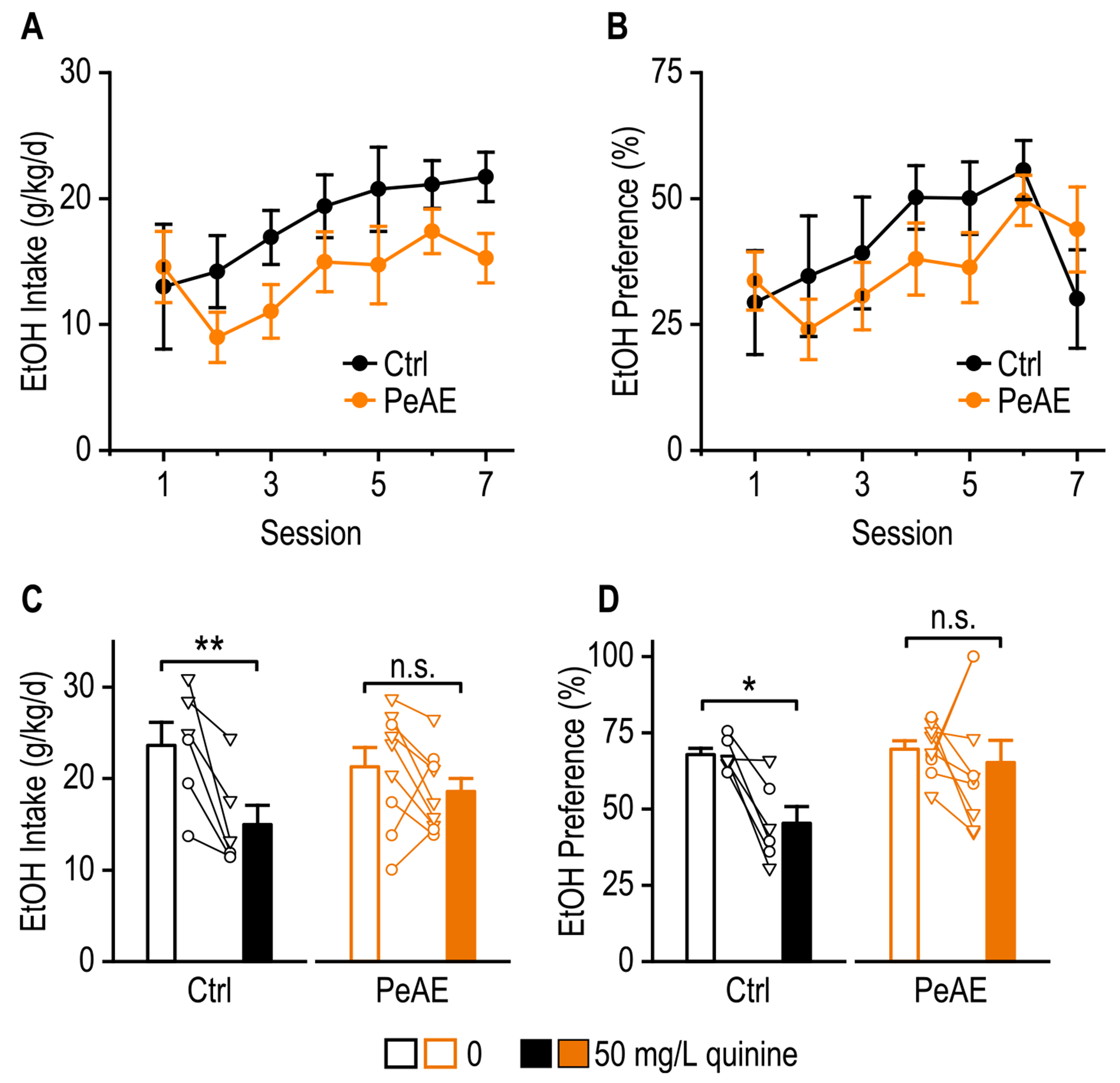
PeAE reduces sensitivity to quinine-adulterated alcohol. PeAE and Ctrl ChATCre; Ai14 mice (~6 months old) were trained to consume alcohol using an intermittent-access two-bottle choice procedure with 20 % alcohol. ***A***, PeAE did not affect acquisition of alcohol consumption. Group × session interaction, *p* = 0.544 by GLMM ***B***, PeAE had no effect on preference for 20 % alcohol. Group × session interaction, *p* = 0.33 by 2-way RM ANOVA. ***C***, Ctrl, but not PeAE mice, significantly reduced alcohol intake when 50 mg/L quinine was added. Group x session effect, *p* = 0.070, by 2-way RM ANOVA with Tukey’s post-hoc test, **p < 0.01. ***D***, Preference for alcohol was significantly decreased by 50 mg/L quinine in Ctrl mice but remained unchanged in PeAE mice. Group x session effect, *p* = 0.11 by 2-way RM ANOVA with Tukey’s post-hoc test, *p < 0.05. n = 6 mice (3 females, 3 males) from 4 litters (Ctrl) and 9 mice (5 females, 4 males) from 5 litters (PeAE). Triangle symbols are female data points and circles are male data points.

Subjects were randomly assigned to experimental conditions in all experiments to reduce selection bias. Experimenters were blinded to group identity during data collection and analysis for histological quantification (Fig. 1), instrumental reversal learning (Fig. 4), and alcohol/quinine drinking behavior (Fig. 5). In contrast, blinding was challenging for slice electrophysiology (Fig. 2) and live-tissue confocal imaging (Fig. 3), as the same individuals conducted animal handling, surgeries, data acquisition, and analysis. To minimize bias, these experiments were performed using standardized acquisition protocols and pre-defined analysis parameters. Electrophysiology and imaging were also conducted in a pairwise manner to control for variability across solution batches and recording days.

## 3. Results

### 3.1. PeAE reduces numbers of ChAT^+^ neurons in the posterior striatum

To determine the impact of PeAE on the number of CINs, pregnant Ai14 (Cre-dependent tdTomato) dams were exposed to 2BC drinking or water and crossed with male ChAT-Cre sires to produce PeAE or Ctrl ChAT-Cre; Ai14 offspring, respectively (Cheng et al., 2018). Mice from both groups were perfused at ~8 months of age. Coronal sections from ChAT-Cre; Ai14 Ctrl and PeAE mice were prepared and stained for choline acetyltransferase (ChAT) to label neurons expressing ChAT at the time of perfusion. Sections were imaged using confocal microscopy, and both ChAT-positive (ChAT^+^) and tdTomato-positive (tdT^+^) neurons in the striatum were quantified across three anterior-posterior levels: anterior (Ant.,AP: 0.90 ± 0.04), posterior (Post.,AP: 0.09 ± 0.06), and tail (Tail, AP: 1.13 ± 0.05). We observed a significant reduction in ChAT^+^ neuron number within the posterior striatum of PeAE mice compared to controls, with no differences in the anterior or tail regions (Fig. 1G: group x level, *F*_(2,12)_ = 3.67, *p* = 0.057 by 2W RM ANOVA; Tukey’s post-hoc tests: Ant: *p* = 0.235, Post: *p* = 0.043, Tail: *p* = 0.841). In contrast, tdT^+^ numbers did not differ significantly between groups (Supplementary Fig. 2O). This may be due to the fact that tdT^+^ expression in ChAT-Cre; Ai14 mice marks all neurons with a history of ChAT expression, including those that may no longer express ChAT in adulthood, thereby reducing the specificity of this label for mature, ChAT-expressing neurons during adulthood. We then analyzed ChAT^+^ populations in striatal sub-regions of the posterior plane and found that CIN numbers in the dorsomedial striatum (DMS) and dorsolateral striatum (DLS) were significantly lower in the PeAE than Ctrl mice (Fig. 1H, DLS: *t*_(8)_ = 2.39, *p* = 0.044; DMS: *t*_(8)_ = 2.46, *p* = 0.039). We do note that when analyzed by litter, reductions in ChAT^+^ neuron counts within the dorsolateral and dorsomedial striatum were only marginally significant (DLS: *t*_(7)_ = 2.13, *p* = 0.071; DMS: *t*_(7)_ = 2.01, *p* = 0.085, Supplementary Table 1), despite being significant in animal-based analysis. Overall, these results indicate that PeAE significantly reduces the number of CINs in the posterior striatum of adult offspring.

### 3.2. PeAE and PAE reduces CIN firing activity in the DMS of adult offspring

We next investigated whether PeAE affects the function of the remaining CINs in the DMS. To address this, ChAT-Cre; Ai14 mice were utilized so that CINs could be easily identified. Dams were prenatally exposed to alcohol or water using the 2BC drinking procedure previously described. DMS slices were prepared from ~8-month-old offspring, and CINs were identified by their Cre-driven expression of tdT (Fig. 2A). Using cell-attached recording, we found that the spontaneous firing frequency of CINs was significantly reduced in PeAE mice compared to the Ctrl group (Fig. 2B and C; *t*(_6_) = 2.63, *p* = 0.039), indicating that PeAE diminishes CIN firing activity in the DMS of adult offspring.

Using our vapor alcohol exposure model, we next assessed whether PAE alters the intrinsic excitability of DMS CINs in ChAT-Cre; Ai32 mice, which express a yellow fluorescent channelrhodopsin reporter, Ai32, selectively in neurons expressing ChAT. Whole-cell patch-clamp recordings were performed on DMS slices from ~5-month-old Ctrl and PAE offspring. A series of 500-ms step currents were injected via the recording electrode to evoke action potentials in CINs (Fig. 2D). We observed that the same current injection elicited a lower firing frequency in the PAE group compared to the Ctrl group (Fig. 2D; Group: *F*_(1,6)_ = 14.910, *p* = 0.008). Consistent with this reduced excitability, we also observed significantly higher rheobase currents in PAE mice compared to controls (Fig. 2F; *t*_(6)_ = − 2.66, *p* = 0.037), indicating greater synaptic input is required to excite CINs in PAE offspring.

CINs exhibit a characteristic burst-pause firing pattern in response to excitatory input. To examine whether PAE affects this dynamic firing pattern, we recorded CIN spontaneous activity in cell-attached mode before, during, and after inducing burst firing with optogenetic stimulation using a series of blue-light pulses. We found that the pause duration, normalized to the baseline inter-spike interval, was significantly reduced in CINs from PAE mice compared to the Ctrl group when given escalating frequencies of optogenetic stimulation. (Fig. 2G and H: group x simulation, *F*_(2,12)_ = 9.65, *p* = 0.003; 2I: *t*_(6)_ = 2.93, *p* = 0.026). Collectively, these findings suggest that PAE impairs CIN spontaneous and evoked firing in the DMS as well as their characteristic pause in response to burst stimulation.

### 3.3. PAE reduces ACh release in the DMS of adult offspring

Given our findings of disrupted CIN physiology following PeAE and PAE, and considering CINs are the primary source of striatal ACh, we next examined whether PAE alters ACh release in the DMS. A green ACh biosensor, gACh4m, was infused into the DMS of mice exposed to prenatal air (Ctrl) or alcohol vapor (PAE) at ~4 months of age. (Fig. 3A). We then conducted live confocal imaging two weeks later. We found that the amplitude of spontaneous ACh release events was significantly lower in PAE mice compared to Ctrl animals (Fig. 3B and C; *t*_(14)_ = 2.18, *p* = 0.047). However, the frequency of spontaneous ACh release was not significantly affected by PAE (Fig. 3B and D; *t*_(14)_ = − 0.58, *p* = 0.568). These findings suggest that PAE impairs CIN-driven release of ACh in the DMS.

### 3.4. PeAE impairs cognitive flexibility in reversal learning tasks of adult offspring

Previous research demonstrates that DMS CIN activity is integral for behavioral flexibility (Bradfield et al., 2013; Ragozzino et al., 2009), and it is well known that individuals with FASD display persistent cognitive flexibility deficits into adulthood (Green et al., 2009; Mattson et al., 1999; McGee et al., 2008). Given our findings of impaired cholinergic function in PeAE and PAE offspring, we investigated whether PeAE impairs cognitive flexibility in adult offspring using an operant reversal learning paradigm. Mice from both groups, ~8 months old, were first trained to acquire initial action-outcome (A-O) contingencies, with left lever presses delivering purified pellets to one magazine and right lever presses delivering grain pellets to another. Acquisition of this initial A-O contingency was confirmed by an outcome devaluation test (Fig. 4A). During reversal training, reward contingencies were reversed, such that left-port lever presses delivered grain pellets, and right-port lever presses delivered purified pellets. Another devaluation test was conducted in the same manner following four days of reversal training to evaluate if subjects learned the new A-O contingencies. (Fig. 4B).

Both groups had similar lever-press rates during initial training, with no significant interaction between group and session (Fig. 4C; *F*_(10,100)_ = 0.62, *p* = 0.80). PeAE did not affect the formation of the initial A-O contingencies, as PeAE and Ctrl mice showed comparable sensitivity to devaluation in the first devaluation test (Fig. 4D; *F*_(1,10)_ = 0.24, *p* = 0.635). Furthermore, the devaluation index did not differ between Ctrl and PeAE mice during this first test (Fig. 4E*; t*_(10)_ = − 1.04, *p* = 0.324). However, after four sessions of reversal training (Fig. 4F), the PeAE group was significantly less sensitive to the second devaluation compared to Ctrl subjects. Ctrl mice significantly reduced their pressing for the devalued lever, while PeAE mice pressed similarly for both the valued and devalued levers (Fig. 4G; *F*_(1,10)_ = 5.76, *p* = 0.037). Consistent with this finding, the devaluation index of the PeAE group was significantly lower than that of the Ctrl group during this second devaluation test (Fig. 4H; *t*_(10)_ = 2.89,*p* = 0.016). It should be mentioned that the group × session interaction was no longer significant when analyzed by litter, however, PeAE litters still showed a significantly lower devaluation index compared to controls (Supplementary Table 1). These findings suggest that PeAE impairs instrumental behavioral flexibility in adult offspring, as evidenced by reduced sensitivity to the second devaluation following reversal learning. The selective deficit observed after reversal learning indicates that PeAE-associated cognitive impairments are specifically related to cognitive flexibility, rather than generalized learning deficits.

### 3.5. PeAE promotes compulsive alcohol consumption in adult offspring

Having found that PeAE offspring exhibit deficits in reversal learning, we next investigated whether PeAE mice show other relevant deficits in behavioral flexibility. A cardinal feature of alcohol use disorder (AUD) in humans is compulsive consumption and seeking of alcohol (Koob and Volkow, 2010). Furthermore, those with FASD show increased susceptibility to developing substance use disorders (Baer et al., 1998; Goldschmidt et al., 2019). Thus, we investigated whether PeAE affected voluntary alcohol drinking in adult offspring. To this end, PeAE and Ctrl ChAT-Cre; Ai14 mice, aged ~6 months old, underwent an intermittent-access to two-bottle choice procedure with 20% alcohol, a frequently used model of AUD. Surprisingly, we found no significant differences between Ctrl and PeAE mice in alcohol intake (Fig. 5A; *F*_(1,13)_ = 2.89, *p* = 0.113) or preference (Fig. 5B; *F*_(1,13)_ = 0.47,*p* = 0.507).

We next hypothesized that PeAE mice may specifically show inflexible, compulsive alcohol drinking behavior. To evaluate this, we adulterated the alcohol solution with 50 mg/L quinine, a bitter compound typically causing aversion and reduced alcohol intake. Although the effect of group x session was marginally significant (Fig. 5C; *F*_(1,13)_ = 3.91, *p* = 0.070), post-hoc analyses revealed mice within the Ctrl group significantly reduced their drinking while PeAE mice did not drink significantly less quinine-adulterated alcohol (Fig. 5C; Ctrl: *p* = 0.003; PeAE *p* = 0.178 by Tukey’s post-hoc test). Consistent with this finding, while there was no effect of group x session on alcohol preference (Fig. 5D; *F*_(1,13)_ = 2.90, *p* = 0.112), within the control group there was a significant reduction in preference for alcohol when quinine was introduced, but this aversion was not observed in PeAE mice (Fig. 5D; Ctrl: *p* = 0.0170; PeAE *p* = 0.526 by Tukey’s post-hoc test). Together, these findings indicate that PeAE exposure leads to increased compulsive alcohol drinking, suggesting diminished sensitivity to negative outcomes.

## 4. Discussion

The present study provides compelling evidence that PeAE and PAE induce significant and enduring impairments in CIN populations and cholinergic function in the DMS, associated with deficits in cognitive flexibility and increased compulsive alcohol-drinking behavior in adult offspring. Specifically, we observed marked reductions in the number of striatal CINs in PeAE mice, impaired DMS cholinergic activity in PeAE and PAE offspring, and impaired performance in reversal learning tasks and increased compulsive drinking behavior in PeAE adult offspring. These findings identify disrupted cholinergic signaling in the DMS as a key neurobiological substrate potentially underlying behavioral inflexibility and compulsivity observed in FASD.

In this study, we used two complementary exposure paradigms, perinatal alcohol exposure via voluntary 2BC drinking and prenatal vapor inhalation, to model distinct aspects of human alcohol use. The vapor model produces high, consistent BACs during E11–E15, a key window for striatal CIN neurogenesis (Marin et al., 2000; Chen et al., 2010) and may more directly impair CIN development. In contrast, the 2BC paradigm spans a broader developmental period, modeling more variable and chronic exposure. This paradigm may additionally influence CIN function via cumulative or indirect mechanisms, which models human drinking patterns. Together, these paradigms model different aspects of FASD in humans, better reflecting the heterogeneous exposure patterns observed in human FASD.

A critical finding of this study is the significant reduction in CIN populations within the striatum following PeAE. Although CINs represent a small proportion of striatal neurons, their profound modulatory influence over MSNs and their essential role in shaping striatal network dynamics makes this loss functionally significant. Our results align with previous studies showing vulnerability of cholinergic populations, including those in the basal forebrain and striatum to neonatal alcohol exposure (Smiley et al., 2021).

Beyond reductions in neuronal number, our study also demonstrates that the functional integrity of surviving CINs is compromised following PeAE and PAE. Electrophysiology experiments revealed significant reductions in spontaneous CIN firing rate and diminished intrinsic excitability, indicating that CINs in PeAE and PAE animals require stronger synaptic input to generate firing. Interestingly, this is consistent with previous work from our laboratory demonstrating that adult exposure to alcohol and cocaine also reduced CIN spontaneous firing (Gangal et al., 2023). Additionally, our findings extend previous observations of CIN excitability deficits reported in the dorsolateral striatum following gestational alcohol exposure (Bariselli et al., 2023). The shortened CIN pause response following excitation is consistent with altered burst-pause responses in adult animals exposed to alcohol (Li et al., 2025; Ma et al., 2022) and suggest CINs may show altered responses to salient stimuli in PAE mice. These results highlight DMS CIN dysfunction as particularly relevant to behavioral impairments observed in FASD. Complementing our electrophysiological findings, we observed significantly reduced ACh release within the DMS in PAE offspring. This reduced release of ACh may be due to both the reduction of DMS CIN numbers and lowered CIN action potential rate, as CINs are the principal source of ACh in the striatum.

Our analyses of operant behavior show that PeAE mice exhibit diminished behavioral flexibility, a behavior mediated in part by DMS cholinergic circuitry (Bradfield et al., 2013; Ma et al., 2022; Ragozzino et al., 2009). While initial instrumental A-O contingency learning was unaffected by PeAE, adaptation to new contingencies during reversal learning was disrupted in PeAE offspring. This selective impairment in reversal learning is in agreeance with previous rodent studies showing reversal learning deficits in T-Maze (Lee and Rabe, 1999; Thomas et al., 2004) and operant discrimination responding (Marquardt et al., 2014) following developmental alcohol exposure. Additionally, our observed increase in compulsive alcohol consumption in PeAE offspring aligns with clinical studies showing that PAE in humans is a risk factor for developing symptoms of AUD (Baer et al., 1998; Goldschmidt et al., 2019), which is characterized by compulsive drinking despite negative consequences (Koob and Volkow, 2010). This compulsivity may also be an aspect of result from broader deficits in inhibitory control and decision-making, which is known to be disrupted by PAE (Fryer et al., 2007; Olguin et al., 2021). Such inflexibility is a hallmark of FASD pathology in humans (Green et al., 2009; Mattson et al., 1999; McGee et al., 2008), and our findings implicate DMS cholinergic dysfunction in potentially mediating this critical deficit.

Given the essential role of ACh in modulating MSN activity and mediating behavioral flexibility (Gangal et al., 2023; Huang et al., 2024; Ma et al., 2022), decreased cholinergic signaling likely contributes to the impaired cognitive functions seen in this study. Indeed, reduced cholinergic tone in the DMS has been implicated in diminished flexibility and compromised decision-making processes (Bradfield et al., 2013; Huang et al., 2024; Ma et al., 2022; Ragozzino et al., 2009; Ragozzino et al., 2002), providing a possible mechanistic link between the observed cellular deficits and behavioral impairments. In particular, our finding that the pause response following burst excitation in CINs is shortened by PAE is especially relevant. This finding is consistent with previous work implicating shortened CIN pause responses after adult alcohol exposure with impaired behavioral flexibility (Li et al., 2025; Ma et al., 2022). Pauses in CIN activity are thought to allow for new corti- costriatal plasticity to occur (Reynolds et al., 2022; Zucca et al., 2018), so the shortened CIN pause in PAE mice may hamper the formation of updated corticostriatal plasticity and associated changes in striatal output during salient events when CINs undergo burst-pause firing. This alteration of CIN burst-pause dynamics along with an overall reduction in ACh tone may be responsible for the reversal learning deficits observed in PeAE and PAE mice. The altered CIN pause response may similarly explain the inflexible drinking seen in PeAE offspring, as the salience of quinine is likely to elicit burst and pause firing in CINs, which are known to modulate alcohol drinking (Sharma et al., 2024). Furthermore, the reduction in spontaneous ACh release may also contribute to this phenomena, as DMS MSNs are strongly modulated by ACh and are integral for alcohol drinking behavior (Cheng et al., 2017).

Our findings are broadly aligned with multiple studies finding that the pro-cholinergic supplement choline attenuates cognitive deficits in PAE subjects. Developmental supplementation of choline, a precursor to ACh, has been shown to improve neurocognitive outcomes in both preclinical (Schneider and Thomas, 2016; Waddell and Mooney, 2017) and human studies (Gimbel et al., 2022; Jacobson et al., 2018; Wozniak et al., 2015). While the mechanism by which choline ameliorates PAE-driven cognitive deficits is not known, it is plausible that choline could restore the lowered cholinergic function observed in this study and others (Bariselli et al., 2023; Smiley et al., 2021).Our results highlight the need for further investigation into the therapeutic potential of targeting cholinergic signaling to improve cognitive outcomes in those with FASD.

To ensure our findings are properly interpreted, several limitations should be acknowledged. First, our study was not powered to detect sex differences. Due to limited sample sizes in some experimental groups, male and female subjects were pooled for analysis. Future studies will be designed with sufficient power to determine whether PeAE and PAE differentially affect male and female offspring. Second, alcohol consumption was assessed using a 2BC paradigm over 24-h sessions. While this method effectively captures total voluntary intake and has been widely used in the field, it lacks the temporal resolution offered by lickometers or volumetric sippers. Third, although histological, behavioral, and drinking experiments were conducted with experimenter blinding, blinding was not feasible for electrophysiology and live-confocal imaging due to logistical constraints. To minimize potential bias, these experiments were performed using standardized acquisition protocols and predefined analysis parameters. Finally, while we identified novel cholinergic dysfunction in DMS CINs of PeAE and PAE offspring, additional mechanisms may contribute to the observed behavioral inflexibility. Developmental alcohol exposure has been shown to affect MSN excitability in the posterior DMS (Roselli et al., 2020) and also has effects on excitatory input to MSNs in both the DMS (Cheng et al., 2018) and DLS (Zhou et al., 2012). Striatal dopamine dynamics are also altered by PAE (Zhou et al., 2012) and gestational alcohol in females (Bariselli et al., 2023). While other mechanisms may contribute to the behavioral effects we observed, DMS CINs have strong modulatory effects on surrounding striatal processes including cortico-striatal plasticity (Reynolds et al., 2022; Zucca et al., 2018) and striatal dopamine release (Threlfell et al., 2012). In the future we will examine the implication of our observed cholinergic dysfunction on surrounding striatal circuits involved in behavioral flexibility.

In conclusion, this study identified a novel and relevant deficit in DMS cholinergic circuitry and identified associated cognitive and behavioral impairments in adult PeAE and PAE offspring. The specific reduction in CIN populations and functional impairments in cholinergic signaling highlight striatal cholinergic neurons as a promising target for therapeutic intervention. Future research aimed at restoring cholinergic function or enhancing CIN activity could yield novel approaches to mitigating cognitive deficits and reducing compulsive behaviors associated with FASD.

## Supplementary Material

1

2

## Data Availability

Data will be made available on request.

## Appendix A. Supplementary data

Supplementary data to this article can be found online at https://doi.org/10.1016/j.neuropharm.2025.110627.
