# Supplementary material for: Perinatal and prenatal alcohol exposure impairs striatal cholinergic function and cognitive flexibility in adult offspring
Source: Neuropharmacology. Author manuscript; Available in PMC 2026 Jun 16. (PMC13271524; doi:10.1016/j.neuropharm.2025.110627)
Supplement: 1 [file NIHMS2180917-supplement-1.pdf]

A Perinatal Alcohol Exposure (PeAE): Two-Bottle Choice Paradigm

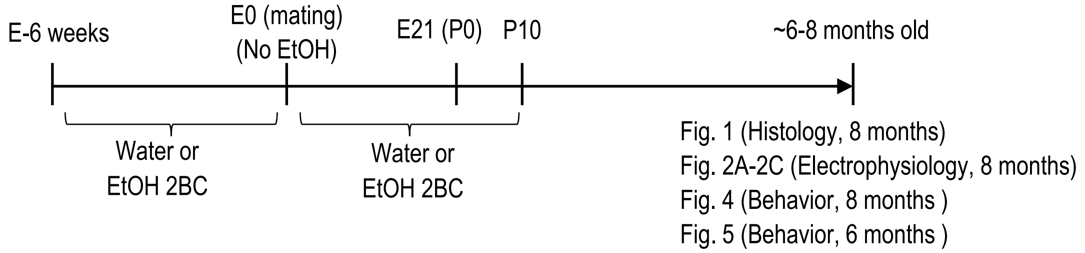

B Prenatal Alcohol Exposure (PAE): Vapor Inhalation Paradigm

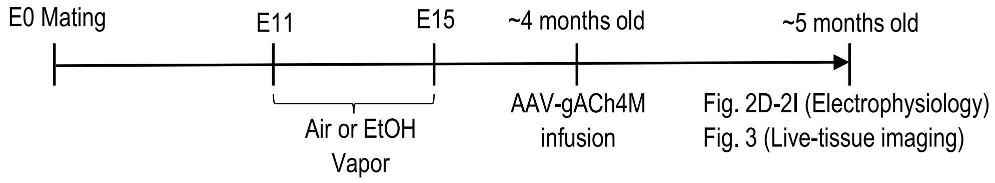

Supplementary Figure 1

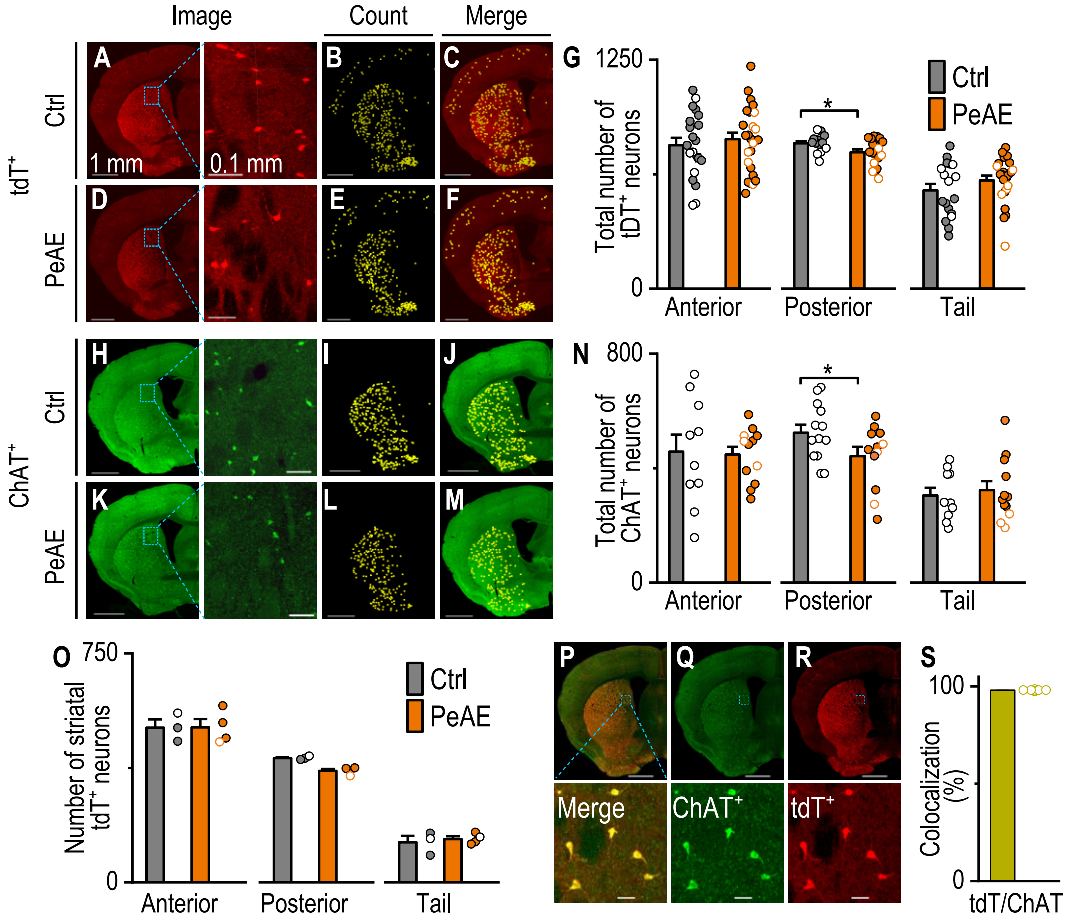

Supplementary Figure 2
