# Supplementary material for: Perinatal and prenatal alcohol exposure impairs striatal cholinergic function and cognitive flexibility in adult offspring
Source: Neuropharmacology. Author manuscript; Available in PMC 2026 Jun 16. (PMC13271524; doi:10.1016/j.neuropharm.2025.110627)
Supplement: 2 [file NIHMS2180917-supplement-2.docx]

## **Supplementary Table 1. Summary of Key Statistical Findings with litter as the experimental unit**

The table shows relevant statistical information including the main text figure each findings is associated with, litter number, statistical comparison performed, group and interaction effects and post-hoc or t-test results when applicable

| Figures | Litter numbers | | Tests | P values | | |
| --- | --- | --- | --- | --- | --- | --- |
|  | Ctrl | PeAE |  | Main Effect (Group) | Interaction  (Group x level) | Tukey Post-hoc or t-tests |
| 1G | 3 | 4 | 2W-RM ANOVA | 0.314 | ^#^0.024 | ^*^0.048, group within posterior level, n.s. at anterior and tail levels |
| 1H-DLS | 4 | 5 | Unpaired t-test | n/a | n/a | 0.071 |
| 1H-DMS | 4 |  | Unpaired t-test | n/a | n/a | 0.085 |
| 4C | 4 | 4 | 2W-RM ANOVA | 0.903 | 0.975 | All PeAE vs Ctrl n.s. |
| 4D | 4 | 4 | 2W-RM ANOVA | 0.924 | 0.553 | All PeAE vs Ctrl n.s. |
| 4E | 4 | 4 | Unpaired t-test | n/a | n/a | 0.156 |
| 4F | 4 | 4 | 2W-RM ANOVA | 0.489 | 0.778 | All PeAE vs Ctrl n.s. |
| 4G | 4 | 4 | 2W-RM ANOVA | 0.436 | 0.078 | All PeAE vs Ctrl n.s. |
| 4H | 4 | 4 | Unpaired t-test | n/a | n/a | ^*^0.025 |
| 5A | 4 | 5 | 2W-RM ANOVA | 0.233 | 0.382 | All PeAE vs Ctrl n.s. |
| 5B | 4 | 5 | 2W-RM ANOVA | 0.733 | 0.575 | All PeAE vs Ctrl n.s.. |
| 5C | 4 | 5 | 2W-RM ANOVA | 0.552 | 0.078 | ^**^0.006 for effect of session within Ctrl. All other PeAE vs Ctrl n.s. |
| 5D | 4 | 5 | 2W-RM ANOVA | ^*^0.014 | ^#^0.025 | ^**^0.003, effect of session within Ctrl. ^***^*<* 0.001, effect of group within quinine session. All other Ctrl vs PeAE n.s. |

^#^*p* < 0.05, **p* < 0.05, ^**^*p* < 0.01, ^***^*p* < 0.001, n.s.: not significant
